# Geroscience insights into difficult-to-treat rheumatoid arthritis: the role of unhealthy aging, comorbidity, and therapeutic complexity

**DOI:** 10.1007/s11357-025-02049-y

**Published:** 2025-12-27

**Authors:** Andrea Lehoczki, Zoltan Ungvari, Ágnes Szappanos, Mónika Fekete, Lilla Gunkl-Tóth, György Nagy

**Affiliations:** 1 Institute of Preventive Medicine and Public Health, Semmelweis University, Budapest, Hungary; 2 Fodor Center for Prevention and Healthy Aging, Semmelweis University, Budapest, Hungary; 3 Doctoral College, Health Sciences Division, Semmelweis University, Budapest, Hungary; 4 Vascular Cognitive Impairment, Neurodegeneration, and Healthy Brain Aging Program, Department of Neurosurgery, University of Oklahoma Health Sciences Center, Oklahoma City, OK USA; 5 International Training Program in Geroscience, Doctoral College/Institute of Preventive Medicine and Public Health, Semmelweis University, Budapest, Hungary; 6 Heart and Vascular Center, Semmelweis University, Budapest, Hungary; 7 Department of Rheumatology and Immunology, Semmelweis University, Budapest, Hungary; 8 Department of Pharmacology and Pharmacotherapy, University of Pécs Medical School, Pécs, Hungary; 9 HUN-REN Chronic Pain Research Group, Pécs, Hungary; 10 Department of Genetics, Cell- and Immunobiology, Semmelweis University, Budapest, Hungary

**Keywords:** Difficult-to-treat rheumatoid arthritis, Aging, Immunosenescence, Inflammaging, Unhealthy aging, Multimorbidity, Frailty, Polypharmacy, Precision medicine, Elderly-onset rheumatoid arthritis, Biological aging, Age-related immune dysfunction, Therapeutic resistance, Personalized therapeutics

## Abstract

Difficult-to-treat rheumatoid arthritis (D2T RA) is an emerging challenge in aging populations, where disease persistence and therapeutic failure often reflect not only autoimmune dysregulation but also the cumulative effects of age-related biological changes across multiple organ systems. This review reframes D2T RA through the lens of geroscience, highlighting how immunosenescence, inflammaging, and organ system vulnerability converge to create a treatment-resistant disease phenotype. Age-associated alterations in adaptive and innate immunity—such as diminished T cell diversity, impaired regulatory function, expansion of age-associated B cells, and heightened inflammasome activation—closely intersect with the immunopathogenesis of RA. The potential contribution of clonal hematopoiesis of indeterminate potential (CHIP) to systemic inflammation and myeloid dysfunction is also discussed as a novel mechanistic link. In parallel, aging of the musculoskeletal system magnifies joint damage, sarcopenia, and pain sensitization. Furthermore, advancing age is also accompanied by multimorbidity, polypharmacy, and frailty, which in turn constrain therapeutic options and increase the risk of adverse events. We argue that D2T RA in the elderly should not be viewed in isolation, but as part of a broader syndemic of age-related diseases driven by shared inflammatory and metabolic pathways. This perspective calls for a shift toward integrated, individualized care strategies that balance efficacy, safety, and quality of life. Future directions include the development of age-adapted treatment guidelines, expanded inclusion of older adults in clinical trials, and the application of artificial intelligence and machine learning to predict high-risk trajectories and personalize management. A geroscience-informed approach offers the conceptual foundation to meet the growing complexity of RA care in aging populations.

## Introduction

Rheumatoid arthritis (RA) is a chronic inflammatory disease characterized by immune-mediated joint destruction and systemic involvement. Despite advances in disease-modifying antirheumatic drugs (DMARDs) and treat-to-target (T2T) strategies [1, 2], a significant proportion—estimated at up to ~30%—remains symptomatic despite standard of care (SOC) therapy, according to the current European Alliance of Associations for Rheumatology (EULAR) recommendations for the management of RA [3, 4]. These individuals are classified as having difficult-to-treat (D2T) RA, a heterogeneous clinical state defined by therapeutic resistance, persistent disease activity, and complex management needs [5]. In older adults, the challenge of D2T RA is further amplified by the interplay between aging biology, multimorbidity, and treatment limitations [6–8].

The aging process profoundly alters immune function, organ resilience, and drug metabolism [9], all of which can modulate disease expression and therapeutic response in RA. Elderly-onset RA (EORA), typically defined as onset after age 65, often presents with atypical features such as prominent large-joint involvement and more acute onset, yet it is also more frequently associated with comorbid conditions, delayed diagnosis, and diagnostic ambiguity [10–13]. Aging not only reshapes the clinical phenotype of RA but also significantly narrows the therapeutic window due to increased frailty, altered pharmacokinetics, and greater susceptibility to adverse events. Moreover, the concept of unhealthy/accelerated aging—characterized by the accumulation of molecular damage, chronic inflammation, and functional decline—exacerbates the course of RA by compounding immune dysregulation, increasing organ system vulnerability, and accelerating the development of comorbidities.

From a geroscience perspective—a field that examines how biological aging processes drive chronic disease [9]—D2T RA in older adults emerges as a multidimensional problem [14]. Age-related immune dysregulation (immunosenescence and inflammaging) contributes to chronic, low-grade inflammation and impaired immune resolution. Simultaneously, aging-associated decline in musculoskeletal integrity, renal and hepatic function, and the accumulation of comorbidities such as cardiovascular disease, osteoporosis, and chronic kidney disease limit treatment options and challenge standard RA management paradigms.

This review explores how aging influences the pathogenesis, clinical course, and therapeutic complexity of D2T RA. We organize these challenges into four interrelated domains: (1) immunosenescence and age-related immune remodeling; (2) aging of target organs and reduced tissue resilience; (3) the syndemic burden of age-related comorbidities; and (4) altered pharmacodynamics and pharmacokinetics in the elderly. By adopting a geroscience-informed lens, we aim to offer a conceptual framework to better understand the emergence of treatment resistance in aging RA patients and to guide more tailored, multidisciplinary strategies for this growing patient population.

## How aging influences RA and D2T risk

The clinical expression and treatment trajectory of RA are deeply influenced by aging-related biological processes. Aging not only alters the immune landscape but also reduces tissue resilience and regenerative capacity, which may lead to a phenotype of RA that is more resistant to standard therapeutic interventions. In older adults, these changes increase the likelihood of entering a D2T state. Two fundamental domains through which aging modifies RA pathogenesis and clinical complexity are immune system dysregulation and musculoskeletal vulnerability.

### Aging and immune system dysregulation: immunosenescence and inflammaging

Aging is accompanied by profound and multifaceted changes in immune function, collectively termed immunosenescence and inflammaging [15]. These processes are not only central drivers of age-related morbidity but also play a critical role in the emergence and persistence of RA in older individuals [16, 17].

#### Cellular and molecular basis of immunosenescence in D2T RA

Immunosenescence refers to the progressive remodeling and decline of the immune system that occurs with aging [15]. This process impacts both the innate and adaptive immune systems, impairing pathogen clearance, reducing vaccine efficacy, increasing cancer risk, and critically altering the regulation of self-tolerance, which predisposes individuals to chronic inflammatory and autoimmune conditions. T and B cell dysfunctions, innate immune changes, dysregulated metabolism, and epigenetic alterations are striking features of immunosenescence [15]. Key features of premature immunosenescence in RA contributing to T cell dysfunction include thymic involution, loss of naïve T cells, expansion of senescent CD28⁻ effector T cells, increased production of proinflammatory cytokines, and accelerated telomere shortening [15–17]. Importantly, these ageing-associated processes appear to be accelerated in RA, contributing to disease onset, progression, and comorbidities [16–18].

T cell precursors are produced and differentiated in the thymus, which reaches its maximum capacity during puberty before gradually undergoing involution, leading to the replacement of thymic tissue with adipose tissue [18]. This change leads to a decrease in naïve T cells, an increase in peripheral late-differentiated memory T cells, and diminished migration of naïve T cells to the periphery [19]. Thymic function can be evaluated by measuring the frequency of peripheral cells that express T-cell receptor excision circles (TRECs) [20]. These are extra-chromosomal DNA sequences that are formed during the rearrangement of T cell receptors. Patients with RA exhibit a lower frequency of TRECs in their peripheral blood mononuclear cells, irrespective of their age, which indicates impaired thymic function [21, 22]. Additionally, the levels of TRECs in CD4 + T cells are significantly reduced in RA patients, resembling the TREC levels found in healthy individuals who are 20 years older [21, 22].

Aging leads to a decrease in T-cell receptor diversity and a loss of expression of the CD28 costimulatory molecule, which is essential for complete T-cell activation and proliferation. There has been a reported increase in the population of CD4 + CD28- and CD8 + CD28- T cells in RA, with a similar accumulation observed in aging [23, 24]. Patients with extra-articular manifestations of RA exhibited higher frequencies of these cells. Additionally, anti-tumor necrosis factor (TNF) therapy significantly reduced the levels of CD8 + CD28- T cells, although it did not affect CD4 + CD28- T cells [25]. This reduction was correlated with clinical improvement, as measured by the disease activity score in 28 joints (DAS28) and C-reactive protein (CRP) levels [26]. In addition, expanded CD4 + CD28- T cells can produce significant amounts of pro-inflammatory cytokines, such as interferon (IFN)-γ and TNF-α. They also express cytotoxic molecules like granzyme B [27]. This phenomenon is associated with chronic immune stimulation and replicative senescence, which can be particularly interesting in cases of EORA, which is characterized by immune aging. It is important to note that the loss of CD28 expression in T cells, often cited as a feature of immunosenescence, also occurs as a consequence of chronic T cell activation, particularly in RA [23, 28–32]. The precise delineation between senescent and activated phenotypes remains debated, and no consensus immunosenescence marker panel currently exists. Recent work by Goronzy and Weyand highlights how metabolic dysregulation and inter-organelle stress responses contribute to T cell abnormalities in RA, some of which may diverge from those seen in physiological aging [16, 33–41]. Thus, while the overlap is conceptually appealing, extrapolation from RA-specific T cell phenotypes to aging requires caution.

Patients with RA develop a unique subset of senescent regulatory T cells (Tregs) characterized as CD4 + FoxP3 + CD28- [20]. These cells exhibit markers of both regulatory T cells and senescent T cells, indicating early signs of aging, such as reduced telomere length and lower levels of TREC. Inflammatory cytokines like TNF-α can induce the formation of these cells. Functionally, senescent Treg-like cells in RA show a diminished ability to suppress the proliferation of effector T cells. Instead, they produce pro-inflammatory cytokines, which disrupt the balance between immune regulation and inflammation [20].

Telomeric erosion represents a notable aspect of premature immunosenescence in RA [42–45]. Research shows that telomeric shortening occurs at an accelerated rate in RA patients, regardless of their age, particularly in granulocytes, peripheral blood mononuclear cells (PBMCs), and CD4 + T cells [21]. The length of telomeres in white blood cells (WBC) is known to decrease progressively by approximately 20 to 40 base pairs per year during healthy aging. In contrast, individuals with RA demonstrate a markedly accelerated rate of telomere attrition, which is 15 times greater than that observed in age-matched healthy controls [46].

Age-associated B cell (ABC)-related immunosenescence, characterized by decreased B cell production and heightened systemic and local inflammatory mediators, is a key factor contributing to B cell dysfunction in immunosenescence [47]. ABC subsets have been identified in patients with RA. These ABCs were found to contribute to the pathogenesis of RA by influencing TNF-α mediated pathways. The presence of increased ABCs was associated with heightened disease activity. Additionally, these elevated ABCs interacted with primary fibroblast-like synoviocytes (FLSs) through TNF-α-mediated ERK1/2 and JAK-STAT1 pathways, leading to increased inflammation in RA [48]. A study also identified interleukin (IL-21) and IFN-γ levels as potential risk factors affecting the presence of ABCs in RA patients [49].

#### Molecular basis of inflammaging and its relevance for D2T RA

Inflammaging, a systemic state of chronic, sterile, low-grade inflammation that plays a pivotal role in promoting both susceptibility to and persistence of RA in older adults [19]. Inflammaging is not driven by overt infection or autoimmunity but arises from the acquisition of an inflammatory phenotype in multiple cell types and the accumulation of damage-associated molecular patterns (DAMPs), cellular debris, and age-related molecular alterations that trigger persistent immune activation. These factors interact with pattern recognition receptors (PRRs)—especially toll-like receptors (TLRs) and NOD-like receptors (NLRs)—on innate immune cells. This engagement triggers intracellular signaling pathways, such as NF-κB, leading to the production of pro-inflammatory cytokines [50]. Cellular senescence plays a crucial role in the process of inflammaging [51, 52]. Senescent cells display a unique characteristic known as the senescence-associated secretory phenotype (SASP), which involves the secretion of various soluble factors [53–57]. These factors include interleukin-1 (IL-1), IL-6, IL-8, IL-13, IL-18, and TNF along with its receptors. This secretion ultimately contributes to the development of the inflammaging phenotype [18, 19]. Immune cell senescence, chronic antigenic stimulation, innate immune activation, and molecular aging pathways are the important drivers of inflammaging in RA. Additionally, tissue-specific senescence in synovial fibroblasts, chondrocytes, and other stromal elements in the RA joint has also been implicated in disease perpetuation and treatment resistance [58–61]. Pro-inflammatory factors released by senescent cells contribute to joint disease progression, systemic comorbidities, and a premature aging phenotype. They amplify and perpetuate the activity of the disease, contributing to the development of RA and associated organ damage [58–61], which is particularly D2T. Importantly, these cells are now direct targets of emerging senolytic and senomorphic therapies, positioning RA as a promising indication for geroscience-guided interventions [61, 62].

#### Clonal hematopoiesis: a novel link between aging and D2T RA pathogenesis

Recent studies suggest that clonal hematopoiesis of indeterminate potential (CHIP)—a common age-associated condition characterized by the expansion of hematopoietic stem cell clones carrying somatic mutations [63, 64]—may also contribute to the pathogenesis or progression of RA [65–67]. CHIP most frequently involves mutations in genes regulating epigenetic modification and inflammation, such as DNMT3A, TET2, and ASXL1, and is strongly associated with aging, affecting over 10–15% of individuals older than 70 [63]. While initially characterized in the context of hematologic malignancies, CHIP has more recently emerged as a risk factor for non-malignant inflammatory diseases [68], particularly cardiovascular disease[69–78], due to its capacity to drive chronic, systemic inflammation [79–82]. CHIP-mutated myeloid cells exhibit enhanced inflammasome activation, increased production of IL-1β and IL-6, and a pro-inflammatory transcriptional profile. These phenotypes closely mirror the inflammatory milieu of RA and may exacerbate synovial inflammation or contribute to systemic inflammatory burden [65, 66]. In murine models, loss of TET2 in hematopoietic cells enhances macrophage activation and increases the severity of inflammatory arthritis, suggesting that CHIP-related mutations may not merely be bystanders but active drivers of immune dysregulation in aging-associated autoimmunity. Although the direct clinical association between CHIP and RA remains underexplored [65, 66], emerging epidemiologic data and cross-sectional analyses from large biobank studies suggest a higher prevalence of CHIP in patients with autoimmune diseases such as Crohn’s disease, psoriasis, vasculitis, and RA [67]. Furthermore, RA is characterized by increased cardiovascular risk and comorbidities, which may be further amplified by CHIP, additionally contributing to the syndromic complexity and treatment resistance observed in elderly RA. From a mechanistic standpoint, CHIP may represent a converging link between immunosenescence, inflammaging, and autoimmunity [16, 65, 67]. The clonal expansion of mutated hematopoietic stem cells skews the composition and function of circulating immune cells—particularly monocytes, macrophages, and neutrophils—toward a more pro-inflammatory and tissue-damaging phenotype. In RA, this could amplify joint inflammation, impair resolution, and possibly reduce responsiveness to immunomodulatory therapies. Given these insights, CHIP seems to contribute to the D2T RA phenotype in aging by promoting both systemic inflammation and resistance to standard treatments. Whether CHIP status may serve as a biomarker for therapeutic stratification or prediction in RA remains an important area for future research. Prospective studies incorporating CHIP screening, longitudinal outcome tracking, and integrative multi-omics approaches are needed to clarify this potentially critical link at the intersection of aging biology and chronic autoimmune disease.

#### Microbiome signatures of aging and D2T RA

Additionally, the aging gastrointestinal tract plays a key role in maintaining a heightened inflammatory state [83–86]. Age-related dysbiosis, characterized by reduced microbial diversity and a shift toward pro-inflammatory bacterial taxa, contributes to increased intestinal permeability. This reduced microbial diversity and compositional shifts can impair immune regulation and increase susceptibility to autoimmune diseases such as RA mediated by the gut-joint axis [87]. RA patients consistently show gut and oral microbiome dysbiosis with the depletion of Gram-negative bacteria and enrichment of Gram-positive bacteria, with reduced diversity and altered abundance of specific taxa (decrease in *Faecalibacterium*, *Fusicatenibacter*, *Enterococcus*, and *Megamonas* and increases in *Eggerthellales*, *Collinsella*, *Prevotella* *copri*, *Klebsiella*, *Escherichia*, *Eisenbergiella*, and *Flavobacterium*) compared to healthy controls [88, 89]_._ Immune dysregulation resulting from microbial imbalance in RA affects both innate and adaptive immunity. An imbalanced microbiota can disrupt innate immunity by causing abnormal activation of pattern recognition receptors, resulting in increased pro-inflammatory cytokines and decreased anti-inflammatory mediators. In adaptive immunity, a dysregulated microbiota contributes to autoimmune responses by affecting antigen-presenting cell function and influencing T cell and B cell activity. Microbiota-induced inflammation can compromise the integrity of tight junctions between intestinal epithelial cells, resulting in enhanced intestinal permeability [90]. This enhanced permeability facilitates the translocation of microbes, their metabolites, and antigenic components into the systemic circulation. Such translocation could significantly impact systemic immune responses and may play a crucial role in the onset and progression of RA [91]. In addition, recent literature indicates that RA-associated gut microbiome dysbiosis treatment with DMARDs could lead to restoration of eubiosis [88]. In the case of microbiota modulations induced by antirheumatic drugs, the immunological correlations of aging and the microbiome should also be considered in the future personalized therapeutic approach of RA. It should be noted that while alterations in microbiome composition have been associated with RA onset, activity, and treatment response, most studies are cross-sectional and causality remains unproven. It is likely that the relationship is bidirectional, with chronic inflammation, immunosenescence, and medication use shaping microbial communities in parallel with potential microbiota-derived immune modulation.

#### Clinical implications of age-related alterations associated with D2T RA

Collectively, immunosenescence drives a state of dysregulated immunity characterized by reduced tolerance, increased autoreactivity, and impaired resolution of inflammation. These alterations may prime the immune system for chronic inflammatory disease and diminish therapeutic responsiveness, while also increasing the risk of adverse outcomes when potent immunosuppression is applied. In RA, these age-related changes help explain why older patients more frequently exhibit persistent disease activity, greater functional decline, and higher rates of treatment failure—hallmarks of the D2T RA phenotype. In clinical practice, immunosenescence also has direct implications for treatment decision-making. Older RA patients exhibit impaired responses to vaccinations, such as pneumococcal or influenza vaccines, and are more vulnerable to infections—a concern that often limits the use of potent immunosuppressive agents such as biologic or targeted synthetic DMARDs. Thus, aging-related immune dysfunction contributes both to disease persistence and to a narrowed therapeutic window, key hallmarks of D2T RA.

In summary, immunosenescence represents a critical intersection of aging biology, innate and adaptive immune dysregulation, and microbial-host interactions. Its impact on systemic and local inflammation, innate and adaptive immune priming, and barrier dysfunction not only contributes to the development of RA in older individuals but also sustains its progression and reduces responsiveness to conventional therapies—making it a key contributor to the D2T RA phenotype in aging populations.

### Aging and target organ vulnerability

While aging impairs immune regulation and fosters chronic inflammation, it also profoundly alters the structural integrity, regenerative capacity, and resilience of target tissues [92]. In the context of RA, these aging-related changes compromise the very organs under autoimmune attack—joints, muscles, and connective tissue [92]—making them more vulnerable to damage and less responsive to therapeutic intervention. This “double burden” of intrinsic tissue fragility and extrinsic immune aggression accelerates disability, reduces treatment efficacy, and contributes to the D2T phenotype in older RA patients.

#### Joint and periarticular tissue degeneration

Aging of articular structures is closely related to osteoarthritis [93]. Joint tissue senescence is marked by cumulative mechanical wear, oxidative stress, and a progressive loss of reparative and regenerative potential. Chondrocyte senescence, decreased proteoglycan synthesis, and increased matrix metalloproteinase (MMP) activity lead to cartilage thinning and loss of elasticity [94–98]. With aging, the cartilage becomes increasingly vulnerable to biomechanical stress and inflammatory stimuli related to autoimmune conditions, which may accelerate tissue degeneration.

At the same time, subchondral bone remodeling becomes dysregulated, often showing features of increased porosity and altered osteoclast-osteoblast coupling. These changes predispose to erosive bone damage and reduce the capacity for joint structural recovery. Aging synovial tissue also undergoes a pro-inflammatory transformation, with increased expression of adhesion molecules, cytokines, and MHC class II, even in the absence of overt RA. This immunological priming further lowers the threshold for destructive synovitis and perpetuates chronic inflammation within the joint microenvironment.

Thus, in older adults, RA develops not in immunologically or mechanically intact tissues, but rather in an already senescent and inflamed environment [99], leading to faster progression of joint destruction and diminished potential for remission or repair [61, 100].

#### Sarcopenia and frailty

In parallel, aging leads to progressive sarcopenia—a decline in skeletal muscle mass, strength, and function—which is exacerbated in RA [101] by chronic inflammation, malnutrition, glucocorticoid use, and physical inactivity [102, 103]. Cytokines such as TNF-α, IL-1β, and IL-6 drive muscle catabolism by activating the ubiquitin–proteasome and autophagy-lysosome pathways, while also inhibiting anabolic signals such as IGF-1. This promotes loss of lean body mass and muscle quality, further impairing mobility and function.

RA-related joint pain and disability reduce physical activity, accelerating the spiral into frailty—a multidimensional syndrome marked by decreased physiological reserve, heightened vulnerability to stressors, and increased risk of falls, hospitalization, and mortality[104–107]. Frailty also correlates with poor treatment tolerance and reduced recovery potential, reinforcing the clinical complexity of D2T RA in older individuals [108, 109]. It is plausible that muscle stem cell (satellite cell) senescence, mitochondrial dysfunction, and impaired neuromuscular junction integrity contribute to the failure of muscle repair observed in aging RA patients, although direct evidence remains limited and warrants further investigation [110–112]. Adverse effects of RA treatments [113–115]—such as glucocorticoid-induced muscle atrophy or mitochondrial toxicity from certain DMARDs—may further exacerbate these deficits. Furthermore, muscle weakness itself may serve as a biomarker of poor prognosis, highlighting the need to integrate musculoskeletal assessment into routine RA care for elderly patients [116].

#### Central sensitization and the amplification of pain and fatigue

Aging also alters the processing and perception of pain, contributing to the subjective burden of disease in ways that complicate clinical assessment. Older adults may experience central sensitization [117], wherein pain signaling pathways become hyperresponsive, amplifying nociceptive input and producing chronic pain in the absence of ongoing peripheral inflammation [118–120]. This phenomenon overlaps with fibromyalgia, osteoarthritis, and other non-inflammatory pain syndromes that are more prevalent with age. In RA, this neuro-sensory alteration can inflate composite disease activity scores, leading to an overestimation of inflammatory disease burden and potentially inappropriate escalation of immunosuppression—particularly in patients whose symptoms are not driven by active synovitis but by altered pain processing.

Similarly, fatigue, a hallmark of both RA [121] and unhealthy aging, is often under-recognized as a complex biopsychosocial symptom involving inflammation, neuroendocrine dysfunction, sleep disturbance, and depression [122–124]. In the elderly, fatigue may be intensified by sarcopenia, anemia, poor nutrition, and comorbid disease, further compounding functional decline and complicating therapeutic decisions.

In summary, the aging of RA target organs—including joints, muscles, and pain-processing pathways—deepens the physiological and functional impact of RA in older adults. This vulnerability compounds the effects of immunosenescence, resulting in a more severe and refractory disease course that defines the D2T RA phenotype in geriatric populations. In this context, D2T RA emerges not simply as a refractory autoimmune disease, but as a manifestation of unhealthy aging, wherein the biological hallmarks of aging amplify both autoimmunity and treatment complexity.

## Aging and comorbidities: a syndemic model

Beyond immune dysregulation and tissue vulnerability, aging introduces a syndemic burden—a clustering of comorbid conditions that interact biologically and socially to exacerbate disease progression, impair therapeutic responses, and increase the risk of adverse outcomes [125–127]. In RA, this syndemic interplay creates a self-reinforcing cycle: comorbidities are not only consequences of chronic inflammation and immunosuppressive therapy but also predisposing factors that amplify RA severity, limit treatment options, and increase the likelihood of entering a D2T state [128]. Older RA patients disproportionately suffer from a range of interrelated conditions—cardiovascular disease, chronic kidney disease, osteoporosis, malignancy, chronic pain syndromes, and infection risk—which independently and synergistically influence the disease trajectory. These comorbidities must be viewed not as background noise but as integral components of the RA phenotype in aging populations, shaping clinical outcomes and necessitating a holistic management paradigm.

### Cardiovascular disease

RA is an independent risk factor for cardiovascular disease (CVD), with a magnitude comparable to that of type 2 diabetes [129–133]. The interplay between RA, aging, and CVD represents a key axis in D2T pathogenesis. This elevated risk is driven by systemic inflammation, which accelerates atherosclerosis through endothelial dysfunction, oxidative stress, lipid modification, and monocyte/macrophage activation [133–136]. Cytokines such as TNF-α, IL-1β, and IL-6 promote plaque formation and instability, while also interfering with reverse cholesterol transport and HDL function. Aging amplifies this process via additional mechanisms, including vascular stiffening, impaired nitric oxide signaling, mitochondrial dysfunction, and increased arterial calcification. Together, these processes create a vascular substrate highly susceptible to inflammatory insult [137]. While mitochondrial dysfunction is a hallmark of aging, we acknowledge that other hallmarks—such as cellular senescence [138] and epigenetic drift [139]—also likely play roles in RA-associated CVD. Importantly, CVD limits therapeutic options in D2T RA. NSAIDs and corticosteroids exacerbate hypertension and fluid retention; JAK inhibitors may increase thrombotic risk; and certain biologics (e.g., IL-6 inhibitors) may alter lipid profiles. Moreover, polypharmacy increases the potential for interactions with antihypertensives, statins, and anticoagulants. Thus, cardiovascular disease is both a driver of RA refractoriness and a barrier to optimal immunomodulation in elderly patients.

### Chronic pain syndromes and psychological comorbidities

Chronic pain is a prevalent and multifactorial problem in older adults with RA [140–142]. Age-related neuroplastic changes in the central nervous system, including impaired descending inhibitory pathways and enhanced nociceptive facilitation, promote the development of central sensitization—a hallmark of fibromyalgia and other non-inflammatory pain syndromes [143]. In RA, the distinction between active inflammatory joint pain and non-inflammatory musculoskeletal pain becomes blurred, especially in older individuals with comorbid osteoarthritis, degenerative spine disease, or fibromyalgia [143, 144].

Psychological comorbidities, such as anxiety, depression, or cognitive decline, are also characteristic of RA, with a higher prevalence in elderly patients, and can also strongly influence disease outcomes [145]. Furthermore, psychosocial factors can also modulate pain perception, resulting in symptom persistence [8, 146]. Beyond its direct impact, chronic pain and psychological comorbidities contribute to physical inactivity and deconditioning. This, in turn, can further aggravate depression, sleep disturbance, and cognitive dysfunction, creating a vicious cycle that ends in symptom persistence and further reduces treatment adherence and worsens functional outcomes.

Altogether, these factors can inflate disease activity scores, leading to misclassification as D2T and prompting unnecessary intensification of immunosuppressive therapy, which may worsen other comorbidities without improving pain [147]. Pain management must therefore be multimodal and include central pain modulation strategies, physical therapy, and non-pharmacologic interventions, potentially also targeting psychological comorbidities in addition to inflammation control [148–154].

#### Infections and immunosuppression risk

Infections represent a major clinical obstacle in older RA patients [155–161]. Aging impairs innate immune surveillance, mucosal barrier function, neutrophil migration, and antigen presentation, while RA itself and especially its treatments further compromise host defense. The use of glucocorticoids, methotrexate, biologics, and JAK inhibitors is associated with an increased risk of opportunistic infections, including herpes zoster, tuberculosis, pneumocystis pneumonia, and bacterial sepsis [162–165]. Immunosenescence contributes to delayed recognition of pathogens, diminished vaccine responsiveness, and prolonged inflammatory responses that cause tissue damage. Furthermore, comorbid conditions such as diabetes and chronic lung disease compound infection risk. In clinical practice, infection risk often restricts the escalation of therapy, particularly in patients with a history of recurrent infections or poor vaccine response. This contributes to therapeutic inertia and under-treatment—a core feature of the D2T RA phenotype in older adults.

#### Osteoporosis and fracture risk

RA and aging both increase the risk of osteoporosis, but via partially distinct mechanisms [166–169]. RA promotes bone loss through systemic inflammation and synovial production of RANKL and TNF-α, which drive osteoclastogenesis [167, 168, 170]. Aging, on the other hand, is associated with declining sex hormones, reduced calcium absorption, impaired osteoblast function, and sarcopenia, all of which contribute to skeletal fragility [171]. Glucocorticoid therapy, even at low doses, remains a key contributor to secondary osteoporosis in RA [168, 171]. This is particularly problematic in older women, who often present with trabecular bone loss, vertebral fractures, and reduced balance and muscle strength, further increasing the risk of falls and catastrophic fractures. Fragility fractures in this population are associated with substantial morbidity, functional decline, institutionalization, and premature death. Importantly, the fear of fractures often limits the use of corticosteroids and other effective anti-inflammatory agents, adding to the complexity of disease management.

#### Chronic kidney disease (CKD)

The prevalence of CKD rises sharply with age and is frequently unrecognized in older RA patients [172, 173]. CKD alters the clearance, metabolism, and toxicity profile of many RA medications, including methotrexate, NSAIDs, JAK inhibitors, and some biologics [174]. Even modest reductions in glomerular filtration rate (GFR) can necessitate dose adjustments or outright avoidance of cornerstone therapies. Inflammation, hypertension, diabetes, and cardiovascular disease—common in aging and RA—contribute to the development and progression of CKD. Furthermore, the kidney itself is an immunologically active organ, and chronic inflammation accelerates glomerulosclerosis, tubular atrophy, and interstitial fibrosis, further limiting renal reserve. Renal impairment also increases the risk of drug-induced toxicity, anemia, electrolyte imbalance, and acidosis, all of which can exacerbate frailty and reduce quality of life. For these reasons, CKD is both a limiting factor in treatment selection and a marker of systemic disease burden in elderly patients with D2T RA.

#### Malignancy and therapy-related risks

Cancer risk increases with age due to genomic instability, impaired immune surveillance, and accumulation of oncogenic mutations. In RA, the risk of certain malignancies—especially lymphoma, is elevated, potentially reflecting both chronic inflammation and immunosuppressive therapy exposure [175–179]. The use of biologics and JAK inhibitors in patients with a history of cancer, or with significant cancer risk factors such as smoking, remains controversial, despite limited evidence for an increased recurrence risk [180–182].. Consequently, many clinicians remain cautious, often opting for less effective or more conservative therapies, which may contribute to persistent disease activity. Current recommendations, including those from EULAR, emphasize an individualized approach to RA treatment in the context of previous or concurrent malignancy [183–185]. Drug selection should consider cancer type, stage, time since remission, and recurrence risk, with close coordination between rheumatology and oncology. Non–TNF biologics such as rituximab or abatacept are often preferred in certain cancer histories, whereas TNF inhibitors and JAK inhibitors are generally avoided during the early years following high-risk cancer diagnoses. In elderly RA, malignancy surveillance is further complicated by non-specific symptoms, overlapping drug toxicities, and impaired physiological reserve. This underscores the importance of oncologic risk stratification, careful pharmacovigilance, and shared decision-making in treatment planning for D2T RA in older adults [181, 183–185].

Collectively, comorbidities in older adults with RA are not isolated conditions but biologically interconnected contributors to disease refractoriness, treatment limitation, and poor outcomes. These conditions interact with age-related immune dysfunction and tissue fragility to create a syndemic environment that sustains inflammation, narrows the therapeutic window, and fosters progression to the D2T RA state. Addressing these comorbidities requires a multidisciplinary, individualized approach, integrating geriatric assessment, risk stratification, and precision pharmacology. Recognizing and targeting this syndemic model is critical to improving outcomes in elderly RA patients facing the dual burden of chronic autoimmunity and aging-related multimorbidity.

## Aging and pharmacological challenges: toward precision therapeutics in D2T RA

The pharmacological management of RA in older adults is complex, owing to aging-related changes in drug metabolism, organ function, and systemic resilience. These alterations collectively reduce the safety margins of immunomodulatory therapy and play a central role in the development of the D2T RA phenotype in geriatric populations. Understanding age-dependent pharmacokinetics and pharmacodynamics is essential for balancing efficacy with safety and for optimizing therapeutic strategies in this vulnerable population [186].

### Altered drug metabolism and clearance

Aging significantly alters the absorption, distribution, metabolism, and excretion of pharmacologic agents [187]. Hepatic and renal function—two primary determinants of drug clearance—decline with age due to physiological and pathological remodeling [188]. Hepatic metabolism, especially phase I reactions mediated by cytochrome P450 (CYP) enzymes, is frequently diminished in older adults. Reduced hepatic blood flow, decreased liver volume, and altered enzyme expression lead to impaired clearance of drugs [189, 190] such as methotrexate [191]. This can result in drug accumulation and heightened risk of hepatotoxicity, particularly in patients with comorbid liver disease or malnutrition.

Similarly, renal clearance declines by approximately 1% per year after the age of 40, even in the absence of overt chronic kidney disease [192]. GFR, tubular secretion, and renal plasma flow are all progressively reduced, impairing the elimination of renally excreted agents. This has major implications for the safe use of methotrexate [193] and biologics, which require dose adjustments or close monitoring. Even low-dose methotrexate—long considered a cornerstone of RA therapy—can become toxic in the presence of age-related renal insufficiency [194]. Age-related changes in body composition also affect drug distribution. Increased fat mass and decreased lean body mass and total body water alter the volume of distribution for lipophilic and hydrophilic drugs, respectively, thereby influencing peak plasma concentrations and half-life. These changes are particularly relevant for corticosteroids and monoclonal antibodies.

### Polypharmacy and drug–drug interactions

Polypharmacy—typically defined as the concurrent use of five or more medications—is exceedingly common among older adults with RA, largely due to the high burden of comorbidities that accumulate with age [195–201]. In this context, the risk of pharmacokinetic and pharmacodynamic interactions rises sharply, often resulting in diminished therapeutic efficacy, unpredictable side effects, or frank toxicity. These interactions can significantly undermine disease control and increase morbidity. For example, the combination of methotrexate and NSAIDs [202]—still frequently prescribed in RA, although current recommendations discourage their routine use—can reduce the renal clearance of methotrexate, thereby increasing the risk of nephrotoxicity and pancytopenia. The nephrotoxic and hematological risks associated with commonly co-prescribed RA medications, including methotrexate and NSAIDs, are exacerbated in the context of age-related decline in renal clearance and polypharmacy-induced interactions [202, 203]. Similarly, glucocorticoids used to control RA flares may interact with antihypertensive agents or diuretics, particularly problematic in elderly patients with pre-existing cardiovascular disease. The use of JAK inhibitors presents another challenge when co-administered with CYP3A4 inhibitors such as certain antifungals or macrolide antibiotics, which can elevate circulating drug levels and intensify immunosuppression beyond the intended therapeutic range [204, 205]. Additionally, targeted therapies, including both biologicals and synthetic drugs, are contraindicated with live vaccines, as they increase the risk of disseminated infection in immunocompromised hosts [206]. The consequences of such interactions are not merely theoretical. Polypharmacy is a well-established contributor to adverse drug reactions (ADRs), which are a leading cause of emergency visits, hospitalizations, functional decline, and mortality among the elderly. These risks are further compounded by age-related factors such as cognitive impairment, frailty, and poor medication adherence, all of which interfere with safe and effective pharmacologic management. In patients with D2T RA, where disease control is already challenging, the presence of polypharmacy can tip the balance further toward therapeutic failure or clinical harm, underscoring the urgent need for individualized medication review and careful risk–benefit assessment.

### A narrower therapeutic window

Due to the aforementioned factors in older patients, the therapeutic window—the range between effective and toxic doses—may be significantly narrower [207–213]. Age-related physiological reserve is reduced across multiple systems, including the bone marrow, liver, kidneys, and cardiovascular system. As a result, the same drug dose that is well tolerated in a younger patient may provoke significant toxicity in an older adult [208–213]. In clinical terms, this necessitates more conservative escalation of therapy, often with lower starting doses, longer titration intervals, and increased vigilance for side effects. This cautious approach, while protective, may contribute to under-treatment, prolonged disease activity, and entry into the D2T RA state. Furthermore, immunosenescence and inflammaging may blunt the efficacy of immunomodulatory agents by altering immune cell responsiveness and cytokine networks, requiring higher doses to achieve the same therapeutic effect—doses that may not be tolerated in an older body.

### The case for individualized pharmacotherapy

The pharmacologic complexity introduced by aging and accelerated aging in RA highlights the need for individualized therapy that moves beyond conventional, one-size-fits-all dosing paradigms [214–217]. As older adults often face a convergence of immunosenescence, reduced organ function, polypharmacy, and comorbidities, the therapeutic window for DMARDs becomes substantially narrower. In this context, a precision-based approach to pharmacotherapy—grounded in the principles of geriatric medicine and personalized care—is essential to ensure both safety and efficacy [218, 219]. One promising strategy is the incorporation of comprehensive geriatric assessment into routine RA management. Evaluating frailty, functional reserve, cognitive capacity, and medication adherence provides critical insight into a patient’s physiological resilience and ability to tolerate aggressive immunosuppressive regimens. Frailty, for example, is not only a predictor of poor outcomes but also an independent risk factor for treatment-related complications, necessitating individualized dose adjustments and supportive interventions. Monitoring of renal and hepatic function must be performed proactively and longitudinally. Likewise, periodic evaluation of liver enzymes is vital in patients receiving hepatically metabolized agents, such as JAK inhibitors, as hepatic clearance may decline silently with age, particularly in the presence of comorbid conditions like nonalcoholic fatty liver disease or polymedication-induced hepatic stress. In selected cases, pharmacogenomic testing may also provide valuable insights [220–223]. Variants in genes involved in drug metabolism, such as those affecting CYP450 enzymes, may explain inter-individual variability in response or toxicity to commonly used RA treatments. While not yet standard of care in rheumatology, this approach may become increasingly relevant for older patients exposed to multiple drugs with narrow therapeutic indices or complex metabolic profiles.

Equally important is the rational use of deprescribing strategies to combat the burden of polypharmacy [195]. Regular review of medication lists, with an eye toward eliminating redundant, non-essential, or high-risk agents, can significantly reduce the likelihood of drug–drug interactions and adverse effects. This process is especially critical in patients whose symptom burden may be misattributed to RA but is in fact the result of drug side effects, such as fatigue, gastrointestinal distress, or cognitive impairment. In this landscape, therapeutic drug monitoring (TDM) emerges as a potential tool to guide personalized dosing. Measuring serum concentrations of methotrexate, biologic DMARDs, or JAK inhibitors can help clinicians achieve a delicate balance: maintaining efficacy while minimizing toxicity [224, 225]. In older patients, for whom over- or underexposure can have serious consequences, TDM may allow for finer titration and adaptive treatment plans based on real-time pharmacokinetic feedback.

## Unhealthy aging as a driver of the D2T RA phenotype

As discussed in the “How aging influences RA and D2T risk,” the “Aging and comorbidities: a syndemic model,” and the “Aging and pharmacological challenges: toward precision therapeutics in D2T RA” sections, multiple aging-related mechanisms intersect to drive the D2T RA phenotype. Here, we synthesize these findings into a unified conceptual framework grounded in the biology of unhealthy aging. We define unhealthy aging as a trajectory of biological aging characterized by cumulative molecular damage, a heightened state of chronic low-grade inflammation, loss of physiological resilience, and increased vulnerability to chronic disease. This trajectory is shaped not only by intrinsic biological processes but also by modifiable environmental and lifestyle exposures—the so-called exposome—including diet quality [226, 227], physical inactivity, obesity [214–217], smoking [228–230], alcohol consumption [231], psychosocial stress, occupational hazards, and environmental pollutants [232–235]. Over time, these factors exacerbate biological aging processes, amplify systemic inflammation, and impair repair mechanisms, thereby increasing susceptibility to age-related diseases such as RA and potentially exacerbating disease severity and treatment resistance in older patients. These biologic alterations—rather than age per se—create a permissive environment for autoimmune persistence and treatment resistance. In this model, D2T RA is not only caused by pharmacological failure or intrinsic disease aggressiveness but also by a consequence of unhealthy aging. Biomarkers of accelerated or unhealthy aging—including epigenetic alterations [236–238], mitochondrial dysfunction [239–243], cellular senescence [244–247], impaired proteostasis [248, 249], and stem cell alterations [250], telomere dysfunction [43, 44]—have been documented in the synovium, blood, circulating immune cells, and musculoskeletal tissues of RA patients, particularly in those with late-onset disease or treatment-refractory phenotypes. These processes converge to undermine immune tolerance, delay inflammation resolution, impair tissue repair capacity, and narrow the therapeutic window—especially in individuals with frailty, multimorbidity, and polypharmacy.

This geroscience-based view of D2T RA carries several clinical and research implications. First, it supports the rationale for biological age-informed risk stratification, integrating parameters such as CHIP status, frailty indices, epigenetic clocks, and senescence-associated signatures to identify patients at higher risk of treatment failure. Second, it underscores the urgency of expanding therapeutic approaches to include interventions that target fundamental aging mechanisms. Lifestyle-based strategies that promote healthy aging may serve as critical adjuncts. These include structured physical activity programs, anti-inflammatory dietary patterns, smoking cessation, optimization of sleep quality, and stress-reduction practices, all of which can modulate systemic inflammation, improve metabolic and cardiovascular health, and enhance physiological resilience. Gerotherapeutics [251]—such as senolytics, NAD⁺ boosters, mTOR inhibitors, or caloric restriction mimetics—may also have adjunctive beneficial value in modulating disease severity and improving treatment responsiveness in elderly RA patients. Recognizing D2T RA as a manifestation of unhealthy aging also calls for a paradigm shift in disease management—from a focus on symptom suppression toward prevention, resilience enhancement, and modulation of shared aging-related pathways across multiple organ systems. This framework highlights the potential utility of longitudinal monitoring of aging-related biomarkers [252, 253] and advocates for clinical trials that better represent older, frail, and biologically aged individuals. As the RA population continues to age, adopting an unhealthy aging model of D2T RA may be essential—not only for improving disease control, but also for preserving autonomy, function, and quality of life in later life.

## Management of D2T RA in elderly patients

### Challenges in D2T RA treatment management in aging population

The management of RA in older adults, particularly those with D2T disease, requires a fundamental reevaluation of therapeutic priorities and paradigms [5, 11, 195, 208, 211]. The conventional T2T strategy—focused on remission or low disease activity through stepwise DMARD escalation—has revolutionized outcomes in younger patients but is less applicable in geriatric populations where frailty, multimorbidity, and reduced physiological reserve complicate both escalation and monitoring [5, 11, 195, 208, 211].

Clinical care is often defined by two opposing risks. Overtreatment may occur when persistent symptoms from non-inflammatory sources, such as osteoarthritis or fibromyalgia, are mistaken for active RA, prompting unnecessary immunosuppressive escalation and avoidable toxicity. Undertreatment, on the other hand, arises from concerns about infections, cytopenias, or organ toxicity, leading to therapeutic inertia and uncontrolled inflammation with irreversible joint damage [5, 11, 195, 208, 211].

Overcoming these challenges requires a shift from a rigid, protocol-driven T2T approach to a more individualized, holistic, and context-sensitive strategy—one that balances efficacy, safety, and patient-centered outcomes. The concept of “T2T with flexibility” has emerged in this regard, advocating for modified treatment goals in the elderly that consider not just inflammatory remission, but also quality of life, physical function, and independence [254].

### Patient-centered and multidisciplinary strategies in elderly D2T RA

Managing elderly patients with D2T RA requires a multidisciplinary, patient-centered model of care. Comprehensive geriatric assessment can help guide risk stratification by evaluating frailty, cognition, comorbidities, and social support. Specialist input—such as cardiology, nephrology, or infectious disease—helps optimize treatment in the context of multimorbidity, while pharmacists play a key role in deprescribing and monitoring interactions.

Shared decision-making is particularly important in this setting. Older adults often value autonomy and function over numerical targets of disease control. Engaging patients and caregivers in goal setting, treatment planning, and risk–benefit discussions leads to more appropriate care and better adherence. Tools such as comprehensive geriatric assessment, frailty scoring systems, and patient-reported outcome measures (PROMs) can support this process and promote precision in care delivery.

Nonpharmacological treatment (NPT) options are essential in RA, especially if there are limited drug options, particularly in the case of elderly patients. NPTs offer safe and effective support in the management of D2T RA [255]. Non-pharmacologic interventions—physical therapy, occupational therapy, structured exercise, pain psychology, and nutritional support—can significantly improve outcomes by enhancing mobility, alleviating pain, and supporting self-management.

Collectively, addressing therapeutic inertia and clinical complexity in aging D2T RA requires a paradigm shift: from disease-focused algorithms to person-centered care that adapts to physiological aging, multimorbidity, and individual patient values. A multidisciplinary, flexible, and proactive approach holds the key to improving outcomes in this vulnerable population.

## Future research directions: toward an age-informed paradigm for D2T RA

As the global population ages, the prevalence of D2T RA in older adults will continue to rise, underscoring the need for an age-informed therapeutic paradigm. Future research must clarify how aging biology shapes treatment response and disease course. The convergence of immunosenescence and inflammaging with autoimmune dysregulation represents a biologically plausible basis for both disease onset and persistence in older individuals. Yet, the precise molecular pathways through which these age-related processes influence RA severity, treatment response, and systemic complications remain incompletely understood. Deeper insights into these mechanisms—spanning T and B cell remodeling, innate immune priming, and hematopoietic clonal evolution (e.g., CHIP)—may reveal novel therapeutic targets and inform biomarker discovery. In parallel, there is growing recognition that D2T RA is a rather heterogeneous condition and often emerges as part of broader unhealthy aging trajectories, marked by the cumulative burden of multimorbidity, frailty, polypharmacy, and loss of physiological resilience. The same biologic processes—chronic low-grade inflammation, immune senescence, metabolic dysfunction, and organ system vulnerability—underlie not only RA but also other prevalent age-related conditions, such as type 2 diabetes, hypertension, osteoporosis, chronic kidney disease, and cardiovascular disease. These conditions frequently co-occur, interact, and amplify one another, generating a synergistic clinical burden that cannot be adequately addressed by disease-specific approaches.

This syndemic view emphasizes that RA in older adults cannot be managed in isolation but must be understood within the broader biology of aging-related conditions. Comorbid diabetes, cardiovascular disease, malignancy, and renal impairment frequently intersect with RA, while frailty and reduced physiological reserve restrict the safe use of intensive pharmacologic strategies and increase the risk of harm. To meet this challenge, artificial intelligence (AI)- and machine learning (ML)-driven analytic platforms offer a promising path. These tools can integrate high-dimensional data—spanning clinical, molecular, pharmacologic, and social domains—to identify latent patterns, predict which older patients are at risk of entering a D2T trajectory across multiple diseases, and uncover modifiable factors that drive these unhealthy aging profiles. Such tools may also enable more dynamic, individualized treatment planning in the context of constrained physiological reserve.

To translate these scientific insights into clinical progress, age-adapted treatment guidelines and real-world evidence (RWE) analyses are urgently needed. Clinical trials in RA remain poorly representative of older and frail patients, limiting the generalizability of findings. Expanded inclusion criteria, geriatric-specific endpoints, and the use of large registries and cohort studies are essential to bridge this translational gap. Additionally, decision-support tools that incorporate aging biology, functional status, polypharmacy risk, and personal values will be indispensable for shared decision-making.

## Conclusion

The intersection of rheumatoid arthritis and aging represents one of the most complex and urgent challenges in modern rheumatology. D2T RA in older adults is not solely a reflection of refractory inflammation—it is the outcome of converging biological, pharmacological, and systemic aging processes that amplify disease burden and limit therapeutic options. By recognizing aging as a central modifier of disease biology, treatment response, and patient goals, we can move toward a more precise and compassionate care model. This paradigm shift requires multidisciplinary collaboration, integration of geroscience insights, and greater emphasis on real-world diversity among patients. Ultimately, redefining D2T RA through the lens of aging offers not only a path to improved outcomes in this vulnerable population, but also a model for managing other chronic diseases in the context of the aging human system.

## Data Availability

N/A.
